# Characterization of intrahepatic B cells in acute-on-chronic liver failure

**DOI:** 10.3389/fimmu.2022.1041176

**Published:** 2022-11-25

**Authors:** Yudong Zhao, Wei He, Chenchen Wang, Nana Cui, Changjie Yang, Zhengrui You, Bisheng Shi, Lei Xia, Xiaosong Chen

**Affiliations:** ^1^ Department of Liver Surgery, Renji Hospital, School of Medicine, Shanghai Jiao Tong University, Shanghai, China; ^2^ Division of Gastroenterology and Hepatology , Key Laboratory of Gastroenterology and Hepatology, Ministry of Health, State Key Laboratory for Oncogenes and Related Genes, National Health Council (NHC) Key Laboratory of Digestive Diseases, Renji Hospital, School of Medicine, Shanghai Institute of Digestive Disease, Shanghai Jiao Tong University, Shanghai, China; ^3^ Department of Laboratory Medicine, Renji Hospital, School of Medicine, Shanghai Jiao tong University, Shanghai, China

**Keywords:** acute on chronic liver failure, atypical memory B cells, plasma cells, intrahepatic B cells, dysfunction

## Abstract

**Background and objectives:**

Acute on chronic liver failure (ACLF) is characterized by the immunologic dissonance during the prolonged pathogenic development. Both abnormal innate immune response and adaptive T-cell response have been reported in patients with ACLF; however, less is known regarding B cells in ACLF pathogenesis. Previous reports were only based on immunophenotyping of peripheral blood samples. Here, we aim to dissect liver-infiltrating B-cell subpopulation in ACLF.

**Methods:**

Paired liver perfusate and peripheral blood were freshly collected from healthy living donors and recipients during liver transplantation. Liver tissues were obtained from patients with ACLF, cirrhosis, and healthy controls. Flow cytometry was used to characterize the phenotypic and functional alterations in intrahepatic and circulating B-cell populations from ACLF, cirrhosis, and healthy controls. The expression of CD19^+^ and CD138^+^ on liver tissues was examined by immunohistochemistry staining.

**Results:**

In this study, we first deciphered the intrahepatic B cells subsets of patients with ACLF. We found that the ACLF liver harbored reduced fraction of naïve B cells and elevated percentage of CD27^+^CD21^−^ activated memory B cells (AM), CD27^−^CD21^−^ atypical memory B cells (atMBC), CD27^+^IgD^−^IgM^+^(IgM^+^ memory B cells), and CD27^+^CD38^++^ plasma cells than cirrhosis and healthy controls. Moreover, these B subpopulations demonstrated enhanced activation and altered effector functions. Specifically, the ACLF liver was abundant in atMBC expressing higher CD11c and lower CD80 molecule, which was significantly correlated to alanine aminotransferase and aspartate aminotransferase. In addition, we found that intrahepatic CD27^+^CD38^++^plasma cells were preferentially accumulated in ACLF, which expressed more CD273 (PD-L2) and secreted higher granzyme B and IL-10. Finally, the enriched hepatic plasma B cells were in positive association with disease severity indices including alkaline phosphatase and gamma-glutamyl transferase.

**Conclusions:**

In this pilot study, we showed an intrahepatic B-cell landscape shaped by the ACLF liver environment, which was distinct from paired circulating B-cell subsets. The phenotypic and functional perturbation in atMBC and plasma cells highlighted the unique properties of infiltrating B cells during ACLF progression, thereby denoting the potential of B-cell intervention in ACLF therapy.

## Introduction

The term “acute-on-chronic liver failure (ACLF)” was proposed to characterize an acute hepatic insult (1–3). Present with jaundice and coagulopathy, patients with ACLF often complicate within 4 weeks by ascites and/or encephalopathy and result in high mortality (4). Although the specific pathogenesis of ACLF remains to be elucidated, the immunologic dissonance during ACLF development is universally acknowledged (5). A variety of immune cells including NK, CD4^+^T cells, CD8^+^T cells, neutrophils, and myeloid mononuclear cells participate in the clinical course of ACLF by regulating the exquisite balance between pro-inflammatory and anti-inflammatory responses (6–10). Previous research on ACLF mainly focused on innate immune response and adaptive T-cell response (11); thus, B-cell disturbance in ACLF needs further investigation.

B cells orchestrate a crucial component in systemic immune response. Apart from the classic function of antibody and cytokine secretion, distinct B-cell populations exhibit diverse functions in various disease settings (12, 13). For instance, the expansion of CD27^−^CD38^+^ B cells induced CD4^+^ T-cell activation and facilitated HIV infection (14). Traditionally, two markers, CD21 and CD27, were used to dissect blood B cells into four subsets including CD27^−^CD21^+^ B cells (naïve B cells), CD27^+^CD21^+^ B cells [resting memory B cells (RM)], CD27^−^CD21^−^ [atypical memory B cells (atMBC), also known as tissue-like MBC or aged B cells), and CD27^+^CD21^−^B cells [activated memory B cells (AM)] (15). By enriched surface expression of CD86, CD80, HLA-DR, and PD-L1 molecules, CD21^low^ B cells potentially functioned as antigen-presenting cells (APCs) in eliciting immune response (15, 16). In patients with hepatitis B (HBV), virus-specific memory B cells co-expressing coinhibitory receptors were found defective in apoptosis escape and plasma cells differentiation (17).

The alteration in B-cell profile has been explored in various liver diseases, such as non-alcoholic liver disease (18), hepatocellular carcinoma (HCC) (19), chronic hepatitis C virus (20), and hepatitis B virus (HBV) infection (21). Therefore, we could postulate that chronic stimulation may drive B-cell abnormality and can be clinically relevant to disease management. So far, there have been limited studies on B-cell compartment in ACLF. Du et al. previously demonstrated dysregulated B cells in patients with ACLF (22). However, those results were only based on immunophenotyping of peripheral blood samples (23). Here, we isolated ACLF perfusate to comprehensively characterize the landscape of liver-infiltrating B cells. We further determined correlations between different B-cell populations and clinical parameters indicative of liver injury. We deciphered intrahepatic B cells in ACLF and found that these B-cell subpopulations were phenotypically and functionally unique compared to their circulating counterparts. Our findings elucidated the role of intrahepatic B cells in ACLF and provided insight into targeting B cells during disease progression.

## Materials and methods

### Study subjects and samples

A total of 65 study samples (**Supplementary Table 1**) including 34 ACLF (paired sample, n = 18; blood only, n = 8; perfusate only, n = 8) and 14 decompensated liver cirrhosis (DLC) (paired sample, n = 14) during liver transplantation were recruited between 2021 and 2022 in Renji Hospital, School of Medicine, Shanghai Jiao Tong University. Patients with ACLF were diagnosed in accordance with Asian Pacific Association for the Study of the Liver (APASL) ACLF Research Consortium in 2019 (5): patients with compensated cirrhosis (diagnosed or undiagnosed) and those with non-cirrhotic chronic liver disease, who have a first episode of acute liver deterioration due to an acute insult directed to the liver. The criteria were established by (1) jaundice (total bilirubin levels of ≧5 mg/dl); (2) coagulopathy (International Normalized Ratio (INR) of ≧1.5, or prothrombin activity of less than 40%), within 4 weeks by clinical ascites, hepatic encephalopathy, or both. Etiology of enrolled patients with ACLF included HBV infection (n = 25), alcoholic-related liver injury (n = 4), drug-induced liver injury (DILI, n = 1), and autoimmune liver disease (n = 4; primary biliary cholangitis, autoimmune hepatitis, and primary sclerosing cholangitis). The exclusion standards were (1) malignancies, such as HCC; (2) Budd–Chiari syndrome; (3) extra-hepatic portal venous obstruction. Most enrolled patients had accepted artificial liver support. Patients who were admitted with DLC was either biopsy-affirmed or measured by a combination of clinical symptoms, routine laboratory tests, endoscopic examination, type-B ultrasonic, and CT/MRI scan. The enrolled patients with DLC include HBV infection (n = 5) and autoimmune liver disease (n = 9). Seventeen healthy living donors in liver transplantation were used as controls. The demographic and clinical characteristics of the study subjects were described in **Table 1**. The study was conducted and approved by the Ethics Committee of Renji Hospital, Shanghai Jiao Tong University School of Medicine. All enrolled subjects gave written informed consent under the guidance of the Declaration of Helsinki.

**Table 1:** The demographic and clinical characteristics of subjects.

	HC (17)	Cirrhosis (14)	ACLF (34)
Age, years	28 (19-37)	50 (40-66)	48 (14-64)
Gender, male/female(n)	6/11	6/8	23/11
Liver function test			
ALT, U/L	15 (10-35)	34.5 (11-133)	39.5 (12-267)
AST, U/L	18 (15-27)	59 (17-169)	67.5 (27-315)
TBIL, μM	13 (6.5-22.2)	61.4 (14.5-199.8)	303.85 (200.7-827.7)
AKP, U/L	81 (54-129)	184 (61-492)	101.5 (7-536)
γ-GGT,U/L	15 (9-27)	90 (15-511)	37 (10-386)
Albumin, g/L	48 (44.1-54.2)	32.8(25.1-44.3)	33.85 (24.7-47.8)
Creatinine, μM	55 (41-91)	52(34-95)	41.5 (27-900)
Coagulation test			
PT, s	11.4 (10.8-12.5)	14.8 (12.7-18.3)	26.5 (15.4-50.0)
INR	1.01 (0.95-1.12)	1.33 (1.14-1.7)	2.49 (1.41-4.5)
MELD score	NA	14 (9-18)	29 (21-48)
Clinical characteristics			
Ascites, Yes/No(n)	NA	7/7	23/11
Variceal bleeding, Yes/No(n)	NA	7/7	7/27
Hepatic encephalopathy, Yes/No(n)	NA	2/12	18/16
Underlying cirrhosis, Yes/No(n)	NA	14/0	32/2
Bacterial infection, Yes/No(n)	NA	8/6	22/12
Antibiotic use, Yes/No(n)	NA	8/6	22/12
EASL-CLIF criteria, Yes/No(n)	NA	NA	16/18

All values are expressed as median (range).

HC, healthy controls; ACLF, Acute on chronic liver failure; ALT, alanine aminotransferase; AST, aspartate aminotransferase, AKP, alkaline phosphatase.

γ-GGT, γ-glutamyl transpeptadase; TBIL, total bilirubin; PT, Prothrombin time; HBV, hepatitis B virus.

EASL-CLIF, European Association for the Study of the study of the Liver-Chronic Liver Failure; NA, not available.

### Laboratory examinations

All patients and matched cohorts accepted usual laboratory assessments for liver diseases, which include clinical evaluations, complete blood count, liver function tests (AST, ALT, ALP, γ-GGT, Tbil, Dbil, and albumin), renal function (serum creatinine), and coagulation function (international normalized ratio). The tests were performed at the clinical laboratory in Renji hospital, Shanghai Jiao Tong University School of Medicine.

### Immunohistochemistry and confocal staining assay

All liver samples were obtained during orthotropic liver transplantation. For immunohistochemistry (IHC), 10% formalin-fixed and paraffin-embedded liver tissues were cut into 4-μm sections. IHC staining was carried out as previously described (24). Briefly, after heat-mediated antigen retrieval with sodium citrate buffer (pH 6.0) for 20 min and 3% H_2_O_2_ incubation (Beyotime, Shanghai, China, P0100A) for 15 min, liver sections were blocked with 10% goat serum (Solarbio, Beijing, China, SL038) for 30 min at room temperature and then incubated with rabbit anti-human CD19 (Abcam, Cambridge, UK, ab134114; 1:250) or rabbit anti-CD138 (Abcam, Cambrige, UK, ab128936; 1:8,000) overnight at 4°C. After washing in 1× Phosphate Buffered Saline (PBS) (GENOM, Haining, China, GNM20012) for three times, the liver slides were incubated with a horseradish peroxidase–conjugated secondary antibody (Long Island, Shanghai, China, D-3004) at room temperature for 30min and detected by 3,3′-diaminobenzidine (MXB Biotechnologies, Fuzhou, China, MAX007) and imaged by lighted microscope. Liver infiltration of CD19^+^ cells were assessed by counting absolute numbers of CD19^+^ cells per high power 400× field (CD19^+^ number).

Confocal staining assay was performed as mentioned above. After antigen retrieval and 3% H_2_O_2_ incubation, liver sections were blocked with 10% donkey serum (Solarbio, Beijing, China, SL050) for 30 min at room temperature. Then, liver sections were incubated with rabbit anti-human CD19 (Abcam, Cambridge, UK, ab134114; 1:100) and mouse anti-human IgM (Abcam, Cambridge, UK, ab200541; 1:50) or mouse anti-human CD19 (Abcam, Cambridge, UK, ab270715; 1:100) and rabbit anti-human CD11c (Abcam, Cambridge, UK, ab52632; 1:100) overnight at 4°C. After washing in PBS, the sections were incubated with different fluorochrome-conjugated secondary antibodies (Invitrogen, Carlsbad, CA, USA, 1:500) for 30 min at room temperature. Confocal scanning was performed using an LSM-710 laser scanning confocal microscope (Carl Zeiss, Jena, Germany).

### Isolation of human intrahepatic lymphocytes

Intrahepatic lymphocytes (IHLs) were isolated as previously described (25). Briefly, once the organ was excised, liver sections were flushed with saline solutions to remove any residual blood. Small pieces of liver sections were perfused with isotonic fluid supplemented with EDTA (5 mM/L), and then, cells were resuspended in RPMI-1640 medium (Gibco). The suspension was filtered through a 70-μm mesh. After a 5-min centrifugation at 50g, the non-parenchymal hepatic cells in the supernatant were collected and harvested by another 10-min centrifugation at 500g. Subsequently, IHL were further lysed with the Red Blood Cell Lysis Buffer (Sigma) for 3 min. After gentle washing, separated IHLs were labeled with cell surface and intracellular cytokine markers for flow cytometric analysis.

### Flow cytometric analysis

**Figure 1:**
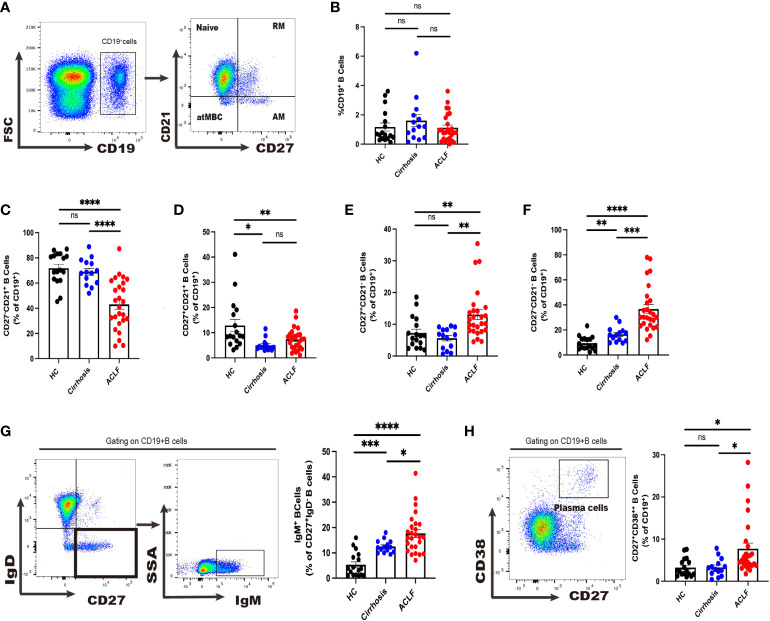
Divergent peripheral B-cell subsets in ACLF, cirrhosis, and HC. **(A, B)** The gating strategy of analysis of CD19+ B cells by flow cytometry and the ratio of CD19+ B cells in blood (HC, n = 17; cirrhosis, n = 14; ACLF, n = 26). **(C–F)** The ratio of CD27−CD21+CD19+ B cells, CD27+CD21+CD19+ B cells, CD27+CD21−CD19+ B cells, and CD27−CD21−CD19+ B cells in blood (HC, n = 17; cirrhosis, n = 14; ACLF, n = 26). (G) The gating strategy for grouping blood CD19+ B cells into IgD−IgM+ subset by flow cytometry and the ratio of IgD−IgM+CD19+ B cells in blood (HC, n = 17; cirrhosis, n = 14; ACLF, n = 26). **(H)** The gating strategy for grouping blood CD19+ B cells into CD2+CD38++ subset by flow cytometry and the ratio of CD27+CD38++CD19+ B cells in blood (HC, n = 17; cirrhosis, n = 14; ACLF, n = 26). *p < 0.05, **p < 0.01, ***p < 0.001, and ****p < 0.0001; ns, not significant.

We obtained peripheral blood mononuclear cells (PBMCs) by a gradient of Ficoll-Paque (GE Healthcare, USA). The freshly enriched PMBCs and IHLs were washed with PBS twice. For intracellular cytokines detection, cells were stimulated in complete RPMI-1640 containing 10% Fetal Bovine Serum (FBS) and Lipopolysaccharide (LPS) (1μg/ml) (Sigma-Aldrich, St. Louis, CA, USA, L2630) plus Leukocytes Activation Cocktail with GolgiPlug (BD Biosciences, San Diego, CA, USA, 550583) in a 37°C humidified CO_2_ incubator for 5 h. Then, cells were stained with live/dead surface markers, fixed with the Fix/Perm kit (BD Biosciences, San Diego, CA, USA, 554714), and incubated with antibodies against intracellular cytokines. The monoclonal antibodies (mAbs) include APC-Cy7–anti-CD19 (BioLegend, USA, Cat#302218); PE-Cy7–anti-CD21 (BioLegend, USA, Cat#354912), PerCP-Cy5-5–anti-CD27 (BD Biosciences, Cat#560612); APC–anti-CD38(BD Biosciences, Cat#555462); PE–anti-IgD (BioLegend, USA, Cat#348204); FITC–anti-IgM (BioLegend, USA, Cat#314506); PerCP-Cy5-5–anti-CCR6 (BD Biosciences, Cat#560467); BV421–anti-CD11c(BD Biosciences, Cat#562561); BV605–anti-CD273 (BD Biosciences, Cat#752600); BV650–anti-CD27 (BD Biosciences, Cat#563228); APC–anti-IgD (BD Biosciences, Cat#561303); BB515–anti-CXCR5 (BD Biosciences, Cat#564624); APC-R700–anti-CD69 (BD Biosciences, Cat# 565154); BV510–anti-CD38 (BD Biosciences, Cat#563251); BV711–anti-IgM (BD Biosciences, Cat#743327); BV786–anti-CD19 (BD Biosciences, Cat#563325); PE-CF594–anti-CXCR3 (BD Biosciences, Cat#562451); PE-Cy7–anti-CD21 (BD Biosciences, Cat#561374); FITC–anti-IL-6 (BD Biosciences, Cat#554544); BV421–anti–TGF-β (BD Biosciences, Cat#562962); BV605–anti-CD38 (BD Biosciences, Cat#562665); BV650–anti-CD80(BD Biosciences, Cat#743866); APC–anti–GM-CSF (BD Biosciences, Cat#502310); Alexa Fluor 700–anti-CD21 (BioLegend, USA, Cat#354918); PE–anti-IL-10 (BD Biosciences, Cat#555088); BV510–anti–TNF-α (BioLegend, USA, Cat#502950); and PE-CF594–anti–granzyme B (BD Biosciences, Cat#562462). All experiments were performed by flow cytometry (Celesta, BD Biosciences and LSRFortessa X-20, BD Biosciences), and data were analyzed with FlowJo software version 10.8 (Tree Star, Ashland, OR USA). Gating strategy was shown in **Figure 1A**.

### Cytometric bead array

Serum cytokines level were quantitated by a BD Human Inflammation CBA (cytometric bead array) kit. Briefly, the standards were prepared by serial dilution according to the manufacturer’s instructions (BD Biosciences, San Jose, CA, USA). The serum samples and standards were incubated with specific capture beads for 2 h at room temperature. The mixture was incubated for additional 1 h with the detection reagent. After a final wash, beads were analyzed on a BD FACSCanto II cytometer. Data analysis was performed using the FCAP Array Software (BD Biosciences, San Jose, CA, USA).

### Statistical analysis

The statistical testing was performed with GraphPad Prism 9.0 (GraphPad Software Inc., USA). Plotted data were expressed as mean ± standard error of mean (SEM). Mann–Whitney *U*-test and paired *t*-test were conducted for continuous variables. Correlations were determined by Pearson or Spearman correlation analysis. One-way analysis of variance (ANOVA) was used for multiple comparisons. All tests were two-tailed, and *P* < 0.05 was considered as statistically significant.

## Results

### Peripheral B-cell subsets in patients with ACLF compared to cirrhosis and HC

First, we sought to examine whether there was a change in CD19^+^ total peripheral B cells and no difference was observed between ACLF, cirrhosis, and healthy control (HC) cohorts (**Figure 1B**). In ACLF, we found a drastic reduction in naïve B cells compared with cirrhosis and HC, indicating that patients with ACLF had more antigen-experienced B cells in blood (p < 0.05 for ACLF vs. cirrhosis, p < 0.001ACLF vs. HC) (**Figure 1C**). No discrepancy was detected in RM between ACLF and cirrhosis (p < 0.05 for ACLF vs. HC and for cirrhosis vs. HC) (**Figure 1D**). Flow Cytometry (FCM) analysis showed an enrichment of AM in ACLF, whereas cirrhosis and HC showed no difference (**Figure 1E**). atMBC, also known as tissue-like memory B cells or aged B cells, were notably expanded in comparison with the remaining two groups (P < 0.001 for ACLF vs. cirrhosis, P < 0.0001 for ACLF vs. HC, P < 0.01 for cirrhosis vs. HC) (**Figure 1F**). We further took advantage of IgM to characterize CD27^+^IgD^−^ IgM^+^ memory B cells (IgM^+^ MBC). Unexpectedly, IgM^+^ memory B-cell subset, accounting for an average of 15% of total B cells, displayed a marked increase compared with liver cirrhosis and HC (**Figure 1G**). We also noticed a marked elevation of CD27^+^CD38^++^ plasma cells in patients with ACLF (p < 0.05 for ACLF vs. cirrhosis and for ACLF vs. HC) (**Figure 1H**). In summary, we managed to provide evidence for altered peripheral B-cell subsets including naïve B cells, AM, atMBC, IgM^+^ MBC, and CD27^+^CD38^++^ plasma cells in patients with ACLF.

### Intrahepatic B-cell populations in ACLF exhibited a unique profile in contrast to cirrhosis and HC

**Figure 2:**
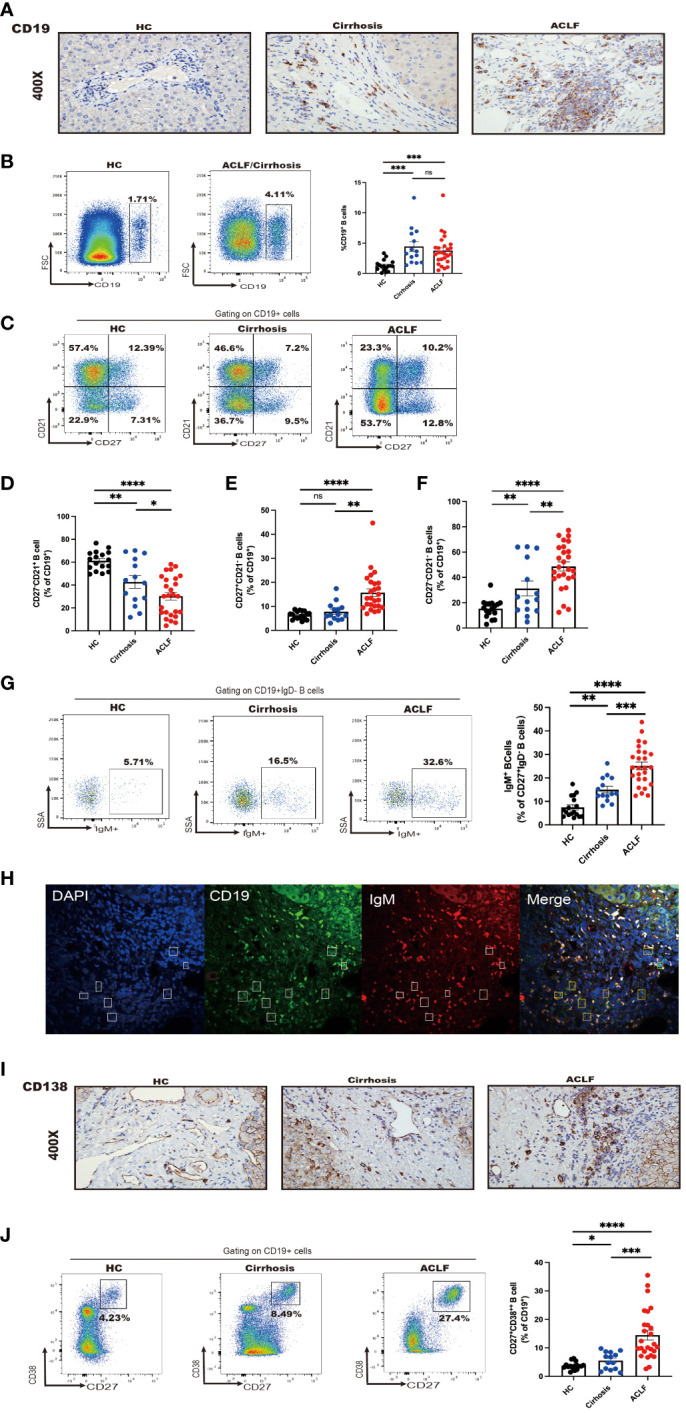
Intrahepatic B-cell delineation in ACLF, cirrhosis, and HC. **(A)** Representative immunohistochemistry images and quantification of CD19-positive cells in liver biopsies from ACLF, cirrhosis, and HC (HC, n = 5; cirrhosis, n = 10; ACLF, n = 10). **(B)** The frequency of CD19^+^ B cells in the liver from patients with ACLF compared with Cirrhosis and HC (HC, n = 17; cirrhosis, n = 14; ACLF, n = 26). **(C–F)** The frequency of CD27^−^CD21^+^CD19^+^ B cells, CD27^+^CD21^−^CD19^+^ B cells, and CD27^−^CD21^−^CD19^+^ B cells in the liver from patients with ACLF compared with cirrhosis and HC (HC, n = 17; cirrhosis, n = 14; ACLF, n = 26). **(G)** The frequency of CD27^+^ IgD^−^IgM^+^CD19^+^ B cells in the liver from patients with ACLF compared with cirrhosis and HC (HC, n = 17; cirrhosis, n = 14; ACLF, n = 26). **(H)** Representative confocal images of IgM^+^CD19^+^ cells using human liver biopsies from patients with ACLF. **(I)** Representative immunohistochemistry images and quantification of CD138-positive cells in liver biopsies from ACLF and cirrhosis (HC, n = 17; cirrhosis, n = 14; ACLF, n = 26). **(J)** The frequency of CD27^+^CD38^++^CD19^+^ B cells in the liver from patients with ACLF compared with Cirrhosis and HC (HC, n = 17; cirrhosis, n = 14; ACLF, n = 26). *p < 0.05, **p < 0.01, ***p < 0.001, and ****p < 0.0001; ns, not significant.

To determine the distinct profile of intrahepatic B cells in ACLF, we next analyzed B-cell subsets in ACLF liver in comparison with cirrhosis and HC. Aside from concurrent abundant lymphocyte liver infiltrate, IHC staining demonstrated an absolute increase in CD19^+^ B-cell number, suggesting accumulated intrahepatic B cells in ACLF (**Figure 2A**). However, cytometry analysis showed no difference in the percentage of infiltrating CD19^+^ B cells within live lymphocytes between ACLF and cirrhosis (**Figure 2B**). In the liver of patients with ACLF, the fraction of naïve B cells was the lowest (**Figures 2C, D**). Hepatic AM was similarly presented in cirrhosis and HC, proposing that this subset might be associated with aberrant liver dysfunction (**Figure 2E**). Liver-infiltrating atMBC appeared highest in ACLF group, followed by liver cirrhosis and healthy donors (**Figure 2F**). Within the intrahepatic IgM^+^ MBC population, we detected that ACLF liver harbored an elevated percentage of IgM^+^MBC compared with cirrhotic patients and HCs (**Figure 2G**). By performing double immunohistochemical staining, we verified the presence of IgM^+^CD19^+^B cells in ACLF liver (**Figure 2H**). In addition, IHC and FACS analysis showed that ACLF liver was specifically rich in CD27^+^CD38^++^plasma B cells (**Figures 2I, J**), raising the possibility that ACLF liver dysfunction might be ascribed to an increase in intrahepatic CD27^+^CD38^++^plasma B cells. In sum, we could conclude that patients with ACLF displayed unique intrahepatic B-cell subsets that were, to some extent, divergent from a cirrhotic setting and physical homeostasis.

### ACLF intrahepatic B-cell populations were distinct from their peripheral counterparts

**Figure 3:**
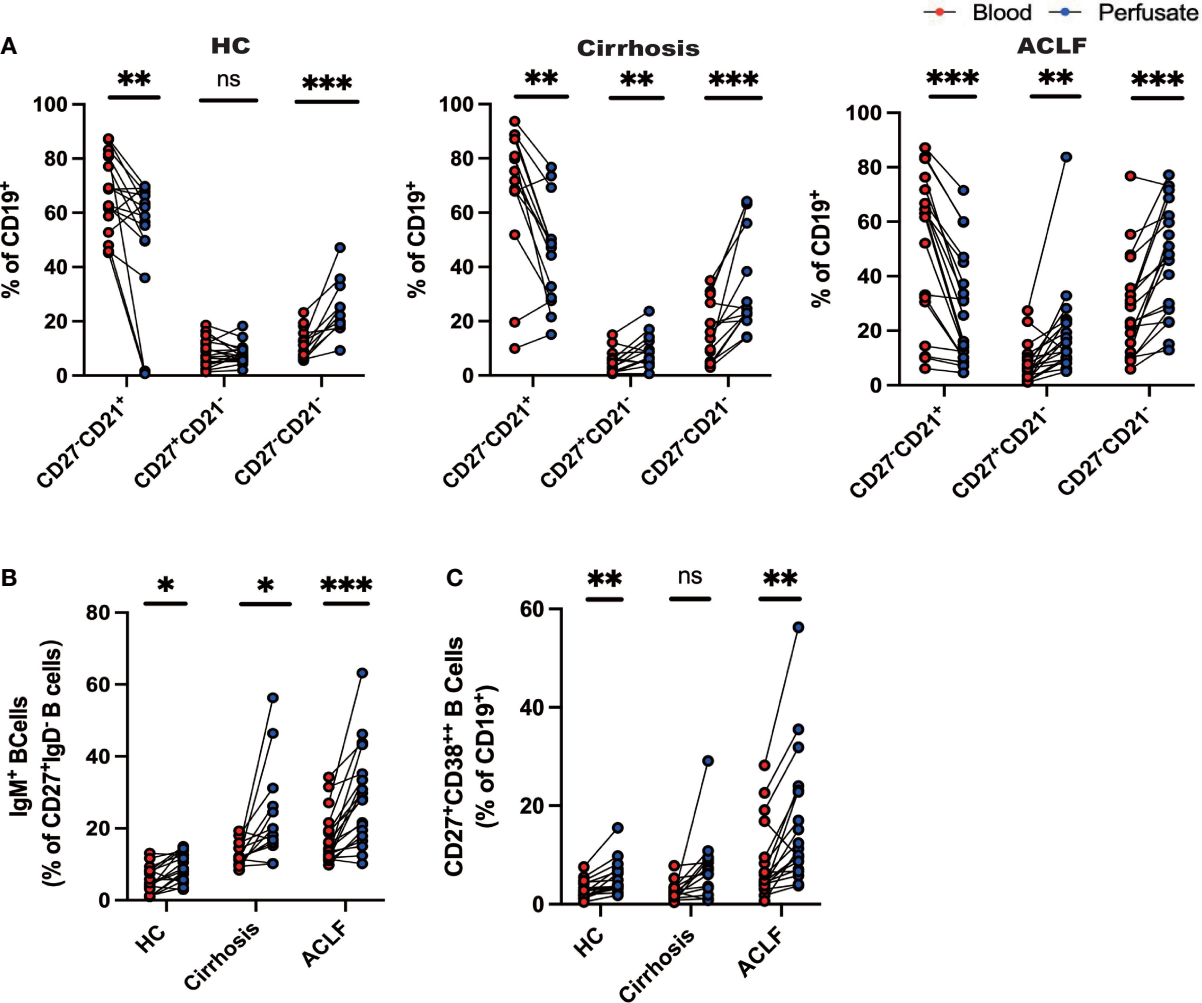
Distinct profile of intrahepatic B cells compared with peripheral blood. **(A)** CD27^−^CD21^+^CD19^+^ B cells, CD27^+^CD21^−^CD19^+^ B cells, and CD27^−^CD21^−^CD19^+^ B cells of liver-infiltrating cell subsets in paired blood and perfusate from ACLF, cirrhosis, and HC (HC, n = 17; cirrhosis, n = 14; ACLF, n = 18). **(B)** CD27^+^IgD^−^IgM^+^CD19^+^ B cells of liver-infiltrating cell subsets in paired blood and perfusate from ACLF, cirrhosis, and HC (HC, n = 17; cirrhosis, n = 14; ACLF, n = 18). **(C)** CD27^+^CD38^++^CD19^+^ B cells of liver-infiltrating cell subsets in paired blood and perfusate from ACLF, cirrhosis, and HC (HC, n = 17; cirrhosis, n = 14; ACLF, n = 18). *p < 0.05, **p < 0.01, and ***p < 0.001; ns, not significant.

Since we have successfully discerned the peripheral B-cell milieus in ACLF from cirrhosis and HC, we set out to investigate whether intrahepatic B-cell populations differed from their peripheral counterparts. In HC liver, we found that the percentage of naïve B cells was reduced compared to peripheral counterparts, whereas atMBC, IgM^+^MBC, and CD27^+^CD38^++^plasma B cells were increased despite no difference in AM ratio (**Figures 3A–C**). In patients diagnosed with cirrhosis, we examined increased fractions of AM, atMBC, and IgM^+^ MBC in cirrhotic liver perfusate (**Figures 3A, B**). However, a similar distribution of CD27^+^CD38^++^plasma B cells between blood and liver was observed (**Figures 3A–C**). To notify, we noticed altered proportions of intrahepatic B cells that can be illustrated by a lower fraction of naïve B cells and a higher fraction of AM and IgM^+^ MBC in ACLF liver (**Figures 3A–C**). Remarkably, we indicated increased percentage of hepatic atMBC and CD27^+^CD38^++^plasma cells in ACLF, which was in sharp contrast to liver cirrhosis and HCs. Thus, we indicated that the discrepancy in intrahepatic and blood B cells in cirrhosis was less diverse compared to ACLF. We verified that intrahepatic ACLF B-cell populations were distinct from peripheral counterparts.

### ACLF intrahepatic B cells pool displayed unique function compared with cirrhosis and healthy controls

**Figure 4:**
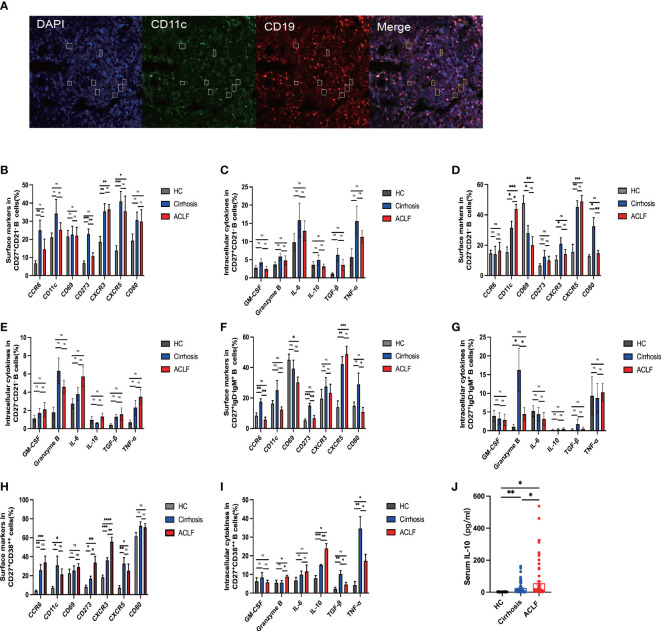
The phenotypic and functional analysis of B-cell subsets in ACLF, cirrhosis, and HC. **(A)** Representative confocal images of CD11c^+^CD19^+^ cells using human liver biopsies from patients with ACLF. **(B, C)** The expression of surface markers and intracellular cytokines on CD27^+^CD21^−^CD19^+^ B cells from the liver of ACLF, cirrhosis, and HC (HC, n = 4; cirrhosis, n = 6; ACLF, n = 7). **(D, E)** The expression of surface markers and intracellular cytokines on CD27^−^CD21^−^CD19^+^ B cells from the liver of ACLF, cirrhosis, and HC (HC, n = 4; cirrhosis, n = 6; ACLF, n = 7). **(F, G)** The expression of surface markers and intracellular cytokines on CD27^+^IgD^−^IgM^+^CD19^+^ B cells from the liver of ACLF, cirrhosis, and HC (HC, n = 4; cirrhosis, n = 6; ACLF, n = 7). **(H, I)** The expression of surface markers and intracellular cytokines on CD27^+^CD38^++^CD19^+^ B cells from the liver of ACLF, cirrhosis, and HC (HC, n = 4; cirrhosis, n = 6; ACLF, n = 7). **(J)** The concentration of cytokines IL-10 in the serums of ACLF, cirrhosis, and HC using cytometric bead array (CBA) (HC, n = 24; cirrhosis, n = 67; ACLF, n = 74). *p < 0.05, **p < 0.01, ***p < 0.001, and ****p < 0.0001; ns, not significant.

Since we have observed difference in intrahepatic B-cell subsets composition, we next investigated the functional change in these subsets. In ACLF, intrahepatic AM expressed lower CD273 (PD-L2) molecule, indicating a decreased interaction of B-T cells and probably denoting an exhausted status. However, no difference was found in the hepatic expression of CCR6, CD11c, CD69, CRCR3, CXCR5, and CD80 in AM of ACLF (**Figures 4B, C**). Among the atMBC subsets, patients with ACLF expressed more CD11c molecules and lower levels of CD80, showing aggravated cell exhaustion and enhanced local chemotaxis (**Figures 4D, E**). We also observed the spatial distribution of CD11c^+^CD19^+^ B cells (**Figure 4A**). IgM^+^ MBC that targeted HBsAg in HBV-ACLF liver was mainly CD27^−^CD21^−^ subset. The reduced percentage of CCR6^+^IgM^+^ MBC in ACLF indicated a diminished tendency of differentiation into antibody-secreting cells (**Figures 4F, G**). Notably, ACLF plasma cells expressed higher level of CD273 (PD-L2) and liver-homing chemokine CXCR3, whereas CXCR5, a lymph node–homing indicator, was similarly expressed between disease and controls (**Figure 4H**). Aside from chemokine receptors, reduced TNF-α^+^ plasma cells indicated that this group of plasma cells alleviated inflammation during ACLF disease progression (**Figure 4I**).We also demonstrated that intrahepatic plasma cells secreted more IL-10 and granzyme B, suggesting the existence of a subset of B cells that could exert regulatory function in ACLF (**Figure 4I**). Moreover, we found heightened level of serum IL-10 in ACLF compared with cirrhosis and HCs (**Figure 4J**). In summary, data from flow analysis indicated that intrahepatic AM, atMBC, IgM^+^ MBC, and plasma cells exhibited altered functions in ACLF.

### Intrahepatic B-cell subsets were associated with clinical parameters in patients with ACLF

**Figure 5:**
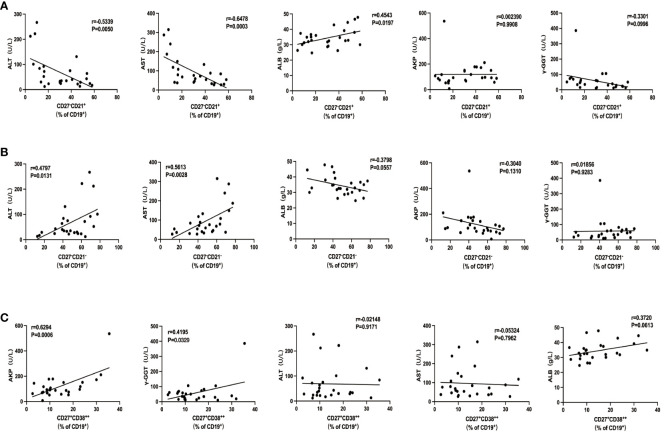
The association of intrahepatic B cells subsets with clinical parameters in patients with ACLF. **(A)** Correlations between the percentage of CD27−CD21+CD19+ B cells and ALT, AST, AKP, γ-GGT, and ALB of patients with ACLF. **(B)** Correlations between the percentage of CD27−CD21−CD19+ B cells and ALT, AST, AKP, γ-GGT, and ALB of patients with ACLF. **(C)** Correlations between the percentage of CD27+CD38++CD19+ B cells and ALT, AST, AKP, γ-GGT, and ALB of patients with ACLF.

To investigate whether these intrahepatic B-cell subsets were reflective of disease severity, we went on to examine the association between those cells with several clinical indices in ACLF. We did not find any association between AM and IgM^+^ MBC with clinical parameters. The percentage of naïve B cells was in an inverse relationship with alanine transaminase (ALT; r = −0.5945, p = 0.0014) and aspartate transaminase (AST; r = −0.6379, p = 0.0005) and was positively correlated with albumin (ALB; r = 0.3828, p = 0.0212) but not with alkaline phosphatase (AKP) or gamma-glutamyl transferase (γ-GGT) (**Figure 5A**). atMBC subset was significantly correlated to ALT (r = 0.5312, p = 0.0052) and AST (r = 0.6525, p = 0.0003) (**Figure 5B**), whereas no correlations were found between atMBC and other clinical indices. Moreover, we determined a significant correlation between CD27^+^CD38^++^ plasma B cells with clinical parameters of liver cholestasis, measured by AKP (r = 0.5526, p = 0.0034) and γ-GGT (r = 0.4556, p = 0.0193) (**Figure 5C**). However, we failed to associate intrahepatic CD27^+^CD38^++^ plasma B cells with ALT, AST, and ALB. Altogether, these findings demonstrated that intrahepatic B-cell lineages could be indicative of liver injury in ACLF.

## Discussion

In ACLF, the composition alterations arise in almost all types of immune cells. Immune defects in ACLF involve abnormalities in functionally reprogrammed innate and adaptive immune cells. Several studies have described the immune perturbations in ACLF. For example, impaired neutrophil function has been attributed to liver injury in ACLF (8). Grønbæk et al. observed an increase in M2-type macrophage markers in the liver of patients with ACLF (26). As a result of continuous immune stimulation from microbial and non-microbial inflammatory signals, patients with ACLF displayed high population of CD14^+^CD15^−^CD11b^+^HLA^−^DR^−^ M-MDSCs (9). In addition, decreased NK cell numbers and a subset of cytotoxic CD56^dim^ CD16^bright^ NK cells were found in the circulation of patients with HBV-ACLF (27). Th17 frequency increased significantly in ACLF, but this is not the case for Treg cell frequency (28). Moreover, Basho et al. demonstrated that enhanced IL-2 secretion altered Tfh signatures and impaired their functionality in advanced liver cirrhosis (29).

Here, we attempted to investigate B-cell composition and functional alteration in patients with ACLF. Peripheral blood immune cells and IHLs were obtained from liver perfusion of patients with ACLF who underwent liver transplantation. We also managed to collect samples from decompensated cirrhosis patients and healthy living donors during liver transplantation (HC) as parallel controls. One limitation in this study is the mismatch of disease cohort in age. To make justifications, a linear statistical model by using R studio was applied to minimize the effect of age variable bias. In our study, patients with ACLF mainly had chronic HBV infection. The etiology of decompensated cirrhosis varied, including chronic HBV infection, autoimmune liver disease (AID) and abuse of alcohol. All patients met the corresponding inclusion criteria of APASL. The ACLF patient cohort in this study was able to reflect the alteration of intrahepatic humoral B-cell immunity in cases of HBV infection.

Initially, we collected peripheral blood samples from patients with ACLF. The flow cytometry analysis indicated differences in naïve B cells, AM, atMBC, IgM^+^ MBC, and plasma cells compared with decompensated cirrhosis patients and HC. We observed similar results in the intrahepatic B-cell pool in ACLF. Accompanied with spontaneous HBV activation, high titer of HBsAg/HBcAg and HBV-DNA in the blood and liver of ACLF initiated naive B cells activation (30). This could be exemplified by a remarkable reduce in both peripheral and intrahepatic naïve B cells in patients with ACLF compared with cirrhosis and HC groups. However, similar effector functions of naïve hepatic B cells were found in three cohorts. The increased intrahepatic AM proportion might be ascribed to abundant antigen exposure and cytokine activation in response to high viral antigen load or inflammatory cascade during ACLF, whereas pathogenic liver environment did not foster AM cytokine secretion.

Alice et al. first put forward a group of intrahepatic viral antigen-specific memory B cells (CD27^−^CD21^−^B cells) known as atMBC, which could facilitate secondary formation of germinal center (GC) B cells and enable plasma cells formation to produce neutralizing antibodies during chronic hepatitis (17). In previous reports, HBsAg-specific B cells appeared more frequent in viral-infected patients but showed impaired ability to mature into anti-HBV plasma cells and fight against viral infection (31). Those atMBC mainly targeted on HBsAg, which was expressed on the hepatocyte surface. atMBC in HBV highly expressed the co-inhibitory receptors PD-1 and FcRL5, showing incomplete signaling pathway and cytokine production, as well as weakened ability to differentiate into mature plasma cells (17, 21, 32, 33). CD11c, a molecule reflecting cell exhaustion and inflammatory-homing ability, along with transcription factor T-bet was expressed on HBV-specific B cells (34, 35). Furthermore, CD11c was expressed in all B-cell lineages despite the differentiation phases. Rubtsov et al. showed that CD11c^+^ T-bet^+^ B cells presented stronger effective Ag-presenting ability than other B-cell subpopulations (36). CD11c^+^ T-bet^+^ B cells were also prone to differentiate into antibody-secreting cells (37). In chronic hepatitis B, the expression of T-bet in atMBC denoted not only its antiviral ability but also an atypical dysfunction phenotype because they were PD-1^high^ (38, 39). The acute and chronic phases of viral infections significantly increased CD27^−^ B-cell population, which could be further expanded by IFN-γ and IL-21 signaling (35, 40, 41). Similarly, our data demonstrated an unexpected accumulation of intrahepatic atMBC, which highly expressed CD11c and CXCR5 and relatively low CD80, indicating an exhausted state with incomplete effector function. We also found an elevated number of intrahepatic CD27^+^IgD^−^IgM^+^B cells, in consistent with a prior study by Le Bert et al. (17).

In our study, we identified enriched proportion of plasma cells in the liver of patients with ACLF, which was positively correlated with clinical indices such as AKP and γ-GGT. We speculated that frequent exposure to HBcAg resulted from massive necrosis of hepatocytes and drove plasma B-cell maturation independent of T helper cells. Moreover, the increased number of tertiary lymphoid structures (TLS) in ACLF was predominantly conducive to plasma B-cell amplification (22). The expansion of CD27^+^CD38^++^ plasma cells in ACLF could also be explained by cytokine stimulation because B-cell activation was, in part, regulated by cytokines. During ACLF, the inflammatory storm can act directly on cytokine receptors and promote the differentiation of plasma cells. IL-6 is an example in case to promote B-cell maturation (42). Heightened level of peripheral IL-21/IL-12 in patients with ACLF directly or indirectly fosters the proliferation of plasma cells with the help of Tfh (22). It remains elusive to assess the effect of Tfh alterations within the B-cell compartment. Indeed, studies have reported both an increase (22, 43) and a decrease (44, 45) in the frequency of circulating Tfh cells, the function of which could be hampered by elevation of IL-2 in advanced cirrhosis (29). However, activated Tfh secreted large amounts of IL-2, favoring the maturation of B-cell responses in the GC (46–48). In this regard, we aim to analyze the functional ability of plasma cells and postulated a group with regulatory function secreting IL-10 and granzyme B in ACLF liver. Inferior secretion of TNF-α by plasma cells compared with liver cirrhosis indicated diminished effector function of plasma cells in ACLF. Therefore, we could hypothesize that the existence of a subset of B cells with regulatory function in ACLF was to control inflammation and avoid tissue damage. In the future, targeting specific B-cell subsets can be a promising treatment of ACLF.

## Data availability statement

The raw data supporting the conclusions of this article will be made available by the authors, without undue reservation.

## Ethics statement

The studies involving human participants were reviewed and approved by Shanghai Jiao Tong University Ethics Committee. Written informed consent to participate in this study was provided by the participants’ legal guardian/next of kin. Written informed consent was obtained from the individual(s), and minor(s)’ legal guardian/next of kin, for the publication of any potentially identifiable images or data included in this article.

## Author contributions

XC, LX, and BS designed the study. YZ, WH, and NC performed the experiments. YZ, CW, and CY collected the samples. YZ analyzed the data. YZ and WH wrote the manuscript. ZY, BS, LX, and XC revised the manuscript. CW obtained the funding. All authors contributed to the article and approved the submitted version.

## Funding

This work was supported by the National Natural Science Foundation of China (NSFC) grants (#81902366 to CW) and Shanghai Yangfan Program (19YF1429000 to CW).

## Acknowledgments

We appreciate all the subjects who provided samples in the study.

## Conflict of interest

The authors declare that the research was conducted in the absence of any commercial or financial relationships that could be construed as a potential conflict of interest.

## Publisher’s note

All claims expressed in this article are solely those of the authors and do not necessarily represent those of their affiliated organizations, or those of the publisher, the editors and the reviewers. Any product that may be evaluated in this article, or claim that may be made by its manufacturer, is not guaranteed or endorsed by the publisher.
