# Corrigendum: Characterization of intrahepatic B cells in acute-on-chronic liver failure

**DOI:** 10.3389/fimmu.2025.1581969

**Published:** 2025-05-02

**Authors:** Yudong Zhao, Wei He, Chenchen Wang, Nana Cui, Changjie Yang, Zhengrui You, Bisheng Shi, Lei Xia, Xiaosong Chen

**Affiliations:** ^1^ Department of Liver Surgery, Renji Hospital, School of Medicine, Shanghai Jiao Tong University, Shanghai, China; ^2^ Division of Gastroenterology and Hepatology, Key Laboratory of Gastroenterology and Hepatology, Ministry of Health, State Key Laboratory for Oncogenes and Related Genes, National Health Council (NHC) Key Laboratory of Digestive Diseases, Renji Hospital, School of Medicine, Shanghai Institute of Digestive Disease, Shanghai Jiao Tong University, Shanghai, China; ^3^ Department of Laboratory Medicine, Renji Hospital, School of Medicine, Shanghai Jiao tong University, Shanghai, China

**Keywords:** acute on chronic liver failure, atypical memory B cells, plasma cells, intrahepatic B cells, dysfunction

In the published article, there was an error in **Figure 1** as published. A single point below 20 in the Cirrhosis group and three points near 80 in the ACLF group were determined to be incorrect. These errors have been corrected, and the updated data points have been incorporated into the figure. Consequently, the corresponding data in **Figures 1D**, **1E**, and **1F** have also been revised. The corrected **Figure 1** and its caption, “Divergent peripheral B cell subsets in ACLF, Cirrhosis and HC,” appear below.

**Figure 1:**
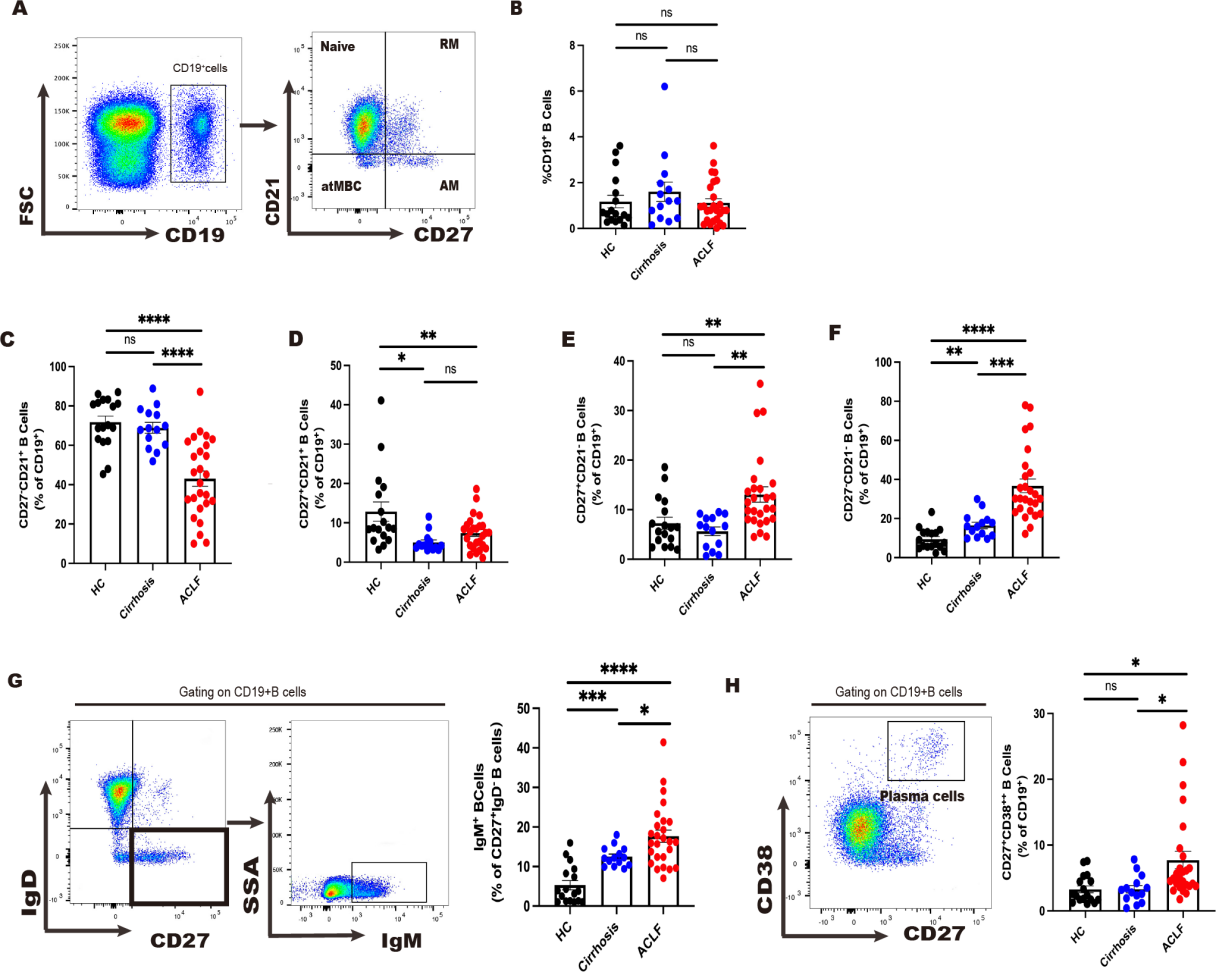
Divergent peripheral B-cell subsets in ACLF, cirrhosis, and HC. (A, B) The gating strategy of analysis of CD19+ B cells by flow cytometry and the ratio of CD19+ B cells in blood (HC, n = 17; cirrhosis, n = 14; ACLF, n = 26). (C–F) The ratio of CD27−CD21+CD19+ B cells, CD27+CD21+CD19+ B cells, CD27+CD21−CD19+ B cells, and CD27−CD21−CD19+ B cells in blood (HC, n = 17; cirrhosis, n = 14; ACLF, n = 26). (G) The gating strategy for grouping blood CD19+ B cells into IgD−IgM+ subset by flow cytometry and the ratio of IgD−IgM+CD19+ B cells in blood (HC, n = 17; cirrhosis, n = 14; ACLF, n = 26). (H) The gating strategy for grouping blood CD19+ B cells into CD27+CD38++ subset by flow cytometry and the ratio of CD27+CD38++CD19+ B cells in blood (HC, n = 17; cirrhosis, n = 14; ACLF, n = 26). *p < 0.05, **p < 0.01, ***p < 0.001, and ****p < 0.0001; ns, not significant.

Furthermore, there were errors in **Figure 5** as published. The erroneous data was in patients with acute-on-chronic liver failure (ACLF). We preliminarily collected all the liver sections from patients receiving liver transplantation and analyzed their liver B cells. we first excluded hepatocarcinoma and then selected patients with high levels of inflammation, mainly based on their total bilirubin levels. As a result, Primary Biliary Cholangitis (PBC) patients were primarily included due to their high jaundice levels. According to the Asian Pacific Association for the Study of the Liver (APASL) diagnostic criteria, ACLF patients should meet with criteria of acute jaundice worsening, coagulopathy, and the presence of extrahepatic organ failure. The coagulation function (PT, INR) of 4 PBC patients did not meet the criteria and generally did not have extrahepatic organ failure, we excluded PBC patients from the ACLF cohort and collected another four Chronic Hepatitis B (CHB)-ACLF liver in our analysis in Figure 2. Notably, the supplementary table related to patient information in the paper is correct and the intergroup comparisons presented in Figure 2 were conducted using this updated ACLF cohort. Regrettably, the analysis of the new ACLF cohort with clinical parameters was not promptly updated in our correlation analysis in **Figure 5**. We have reanalyzed the data and recalculated the p-values and correlation coefficients (R values) in **Figure 5**. The corrected **Figure 5** and its caption, “The association of intrahepatic B cells subsets with clinical parameters in ACLF patients,” appear below.

**Figure 5:**
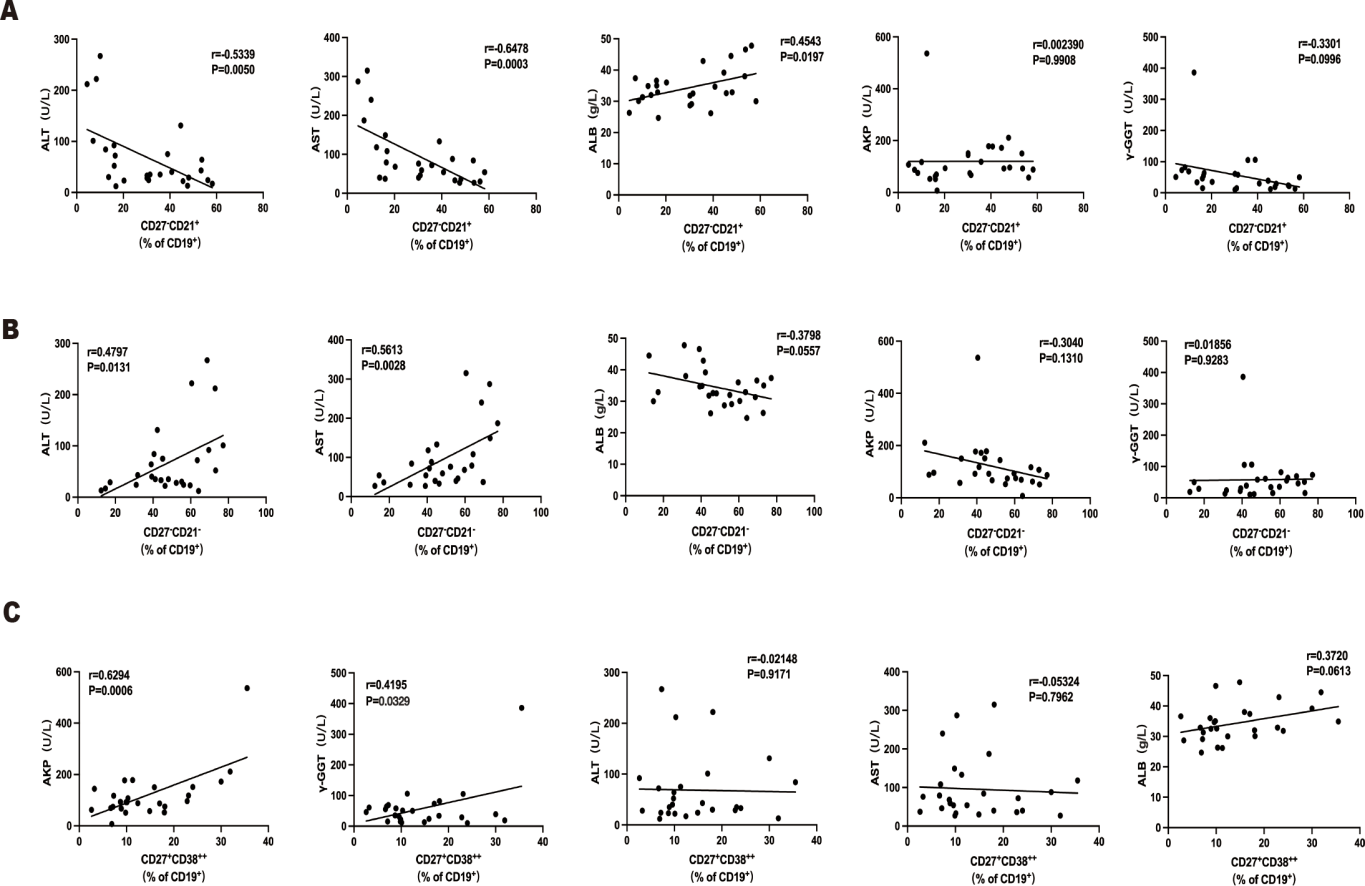
The association of intrahepatic B cells subsets with clinical parameters in patients with ACLF. **(A)** Correlations between the percentage of CD27−CD21+CD19+ B cells and ALT, AST, AKP, γ-GGT, and ALB of patients with ACLF. **(B)** Correlations between the percentage of CD27−CD21−CD19+ B cells and ALT, AST, AKP, γ-GGT, and ALB of patients with ACLF. **(C)** Correlations between the percentage of CD27+CD38++CD19+ B cells and ALT, AST, AKP, γ-GGT, and ALB of patients with ACLF.

And lastly, there were errors in **Table 1**. The demographic and clinical characteristics were incorrectly matched due to adjustments in patient inclusion and exclusion during cohort refinement. The corrected **Table 1** and its caption, “The demographic and clinical characteristics of subjects,” appear below.

**Table 1:** The demographic and clinical characteristics of subjects.

	HC (17)	Cirrhosis (14)	ACLF (34)
Age, years	28 (19-37)	50 (40-66)	48 (14-64)
Gender, male/female(n)	6/11	6/8	23/11
Liver function test			
ALT, U/L	15 (10-35)	34.5 (11-133)	39.5 (12-267)
AST, U/L	18 (15-27)	59 (17-169)	67.5 (27-315)
TBIL, μM	13 (6.5-22.2)	61.4 (14.5-199.8)	303.85 (200.7-827.7)
AKP, U/L	81 (54-129)	184 (61-492)	101.5 (7-536)
γ-GGT,U/L	15 (9-27)	90 (15-511)	37 (10-386)
Albumin, g/L	48 (44.1-54.2)	32.8(25.1-44.3)	33.85 (24.7-47.8)
Creatinine, μM	55 (41-91)	52(34-95)	41.5 (27-900)
Coagulation test			
PT, s	11.4 (10.8-12.5)	14.8 (12.7-18.3)	26.5 (15.4-50.0)
INR	1.01 (0.95-1.12)	1.33 (1.14-1.7)	2.49 (1.41-4.5)
MELD score	NA	14 (9-18)	29 (21-48)
Clinical characteristics			
Ascites, Yes/No(n)	NA	7/7	23/11
Variceal bleeding, Yes/No(n)	NA	7/7	7/27
Hepatic encephalopathy, Yes/No(n)	NA	2/12	18/16
Underlying cirrhosis, Yes/No(n)	NA	14/0	32/2
Bacterial infection, Yes/No(n)	NA	8/6	22/12
Antibiotic use, Yes/No(n)	NA	8/6	22/12
EASL-CLIF criteria, Yes/No(n)	NA	NA	16/18

All values are expressed as median (range).

HC, healthy controls; ACLF, Acute on chronic liver failure; ALT, alanine aminotransferase; AST, aspartate aminotransferase, AKP, alkaline phosphatase.

γ-GGT, γ-glutamyl transpeptadase; TBIL, total bilirubin; PT, Prothrombin time; HBV, hepatitis B virus.

EASL-CLIF, European Association for the Study of the study of the Liver-Chronic Liver Failure; NA, not available.

The authors apologize for this error and state that this does not change the scientific conclusions of the article in any way. The original article has been updated.

